# Graphene oxide/polyvinylpyrrolidone-doped MoO_3_ nanocomposites used for dye degradation and their antibacterial activity: a molecular docking analysis

**DOI:** 10.3389/fchem.2023.1191849

**Published:** 2023-05-09

**Authors:** Muhammad Ikram, Iram Atiq, Alvina Rafiq Butt, Iram shahzadi, Anwar Ul-Hamid, Ali Haider, Walid Nabgan, Francisco Medina

**Affiliations:** ^1^ Solar Cell Applications Research Lab, Department of Physics, Government College University Lahore, Lahore, Punjab, Pakistan; ^2^ Department of Physics, Lahore Garrison University, Lahore, Punjab, Pakistan; ^3^ Punjab University College of Pharmacy, University of the Punjab, Lahore, Pakistan; ^4^ Core Research Facilities, Research Institute, King Fahd University of Petroleum and Minerals, Dhahran, Saudi Arabia; ^5^ Department of Clinical Sciences, Faculty of Veterinary and Animal Sciences, Muhammad Nawaz Shareef University of Agriculture, Multan, Punjab, Pakistan; ^6^ Departament d’Enginyeria Química, Universitat Rovira i Virgili, Tarragona, Spain

**Keywords:** MoO_3_ nanorods, catalytic activity, antibacterial activity, molecular docking, analysis

## Abstract

In this study, MoO_3_ nanostructures were prepared, doped with various concentrations of graphene oxide (2 and 4% GO) and a fixed amount of polyvinylpyrrolidone (PVP) using the co-precipitation method. The motive of this study was to examine the catalytic and antimicrobial efficacy with evidential molecular docking analyses of GO/PVP-doped MoO_3_. GO and PVP were utilized as doping agents to reduce the exciton recombination rate of MoO_3_ by providing more active sites that increase the antibacterial activity of MoO_3_. The prepared binary dopant (GO and PVP)-dependent MoO_3_ was used as an effective antibacterial agent against *Escherichia coli* (*E. coli*). Notably, 4% GO/PVP-doped MoO_3_ showed good bactericidal potential against *E. coli* at higher concentrations in comparison to ciprofloxacin. Furthermore, *in silico* docking revealed the possible inhibitory impact of the synthesized nanocomposites on folate and fatty acid synthesis enzymes, dihydrofolate reductase and enoyl-[acyl carrier protein] reductase, respectively.

## 1 Introduction

A shortage of water resources has occurred internationally as a result of the quick rate of industrialization and the massive growth of the population (Santhosh et al., 2016). Numerous types of waste products are discharged in water, such as pesticides, plastics, textile dyes, and organic and inorganic contaminants, endangering freshwater resources and the ecological environment (Nasrollahzadeh et al., 2021). Heavy metal ions and dyes are two significant categories of aquatic pollutants, and if these substances are present in water, it is no longer good for health. Thoroughly cleaning up the contaminated water can also be challenging (Rafiq et al., 2021). According to the World Bank, dyeing textiles contribute significantly to about 17%–20% of water contamination. Dyes have been used industrially in leather, plastics, printing, cosmetics, and pharmaceuticals (Rafiq et al., 2021). Approximately 7 million metric tons of dyes are produced annually, with over 10,000 commercially accessible dyes (Kapoor et al., 2021). It is challenging to degrade dyes because of their diverse chemical structures, especially when mixed with azo, heterocyclic, and cationic forms (Zhou et al., 2021) Researchers are interested in nanomaterials because of their distinctive physiochemical characteristics and advanced dye-contaminated wastewater treatment techniques (Bari et al., 2022). Small nanostructures (NSs) have splendid surface-to-volume ratios, significantly enhancing chemical (biological and catalytic activity) and physical properties. Among all techniques, the catalytic process has been employed to address the problems of organic impurity (Bari et al., 2022), (Khan et al., 2022). Nano-sized metal oxides (MOs), such as ZnO, MoO_3_, TiO_2_, La_2_O_3_, and CeO_2_, have many applications since they are employed in catalysis and to monitor the antibacterial activity used to degrade industrial wastewater contamination (Ikram et al., 2022). MoO_3_ has attracted attention from different fields, including solar cells, optoelectronic devices, catalysis, electrochromic system dye degradation, oxidative catalysts, antibacterial activities, gas sensors, and photocatalysis. Recent research studies investigated the role of MoO_3_-based materials for antibacterial activities against several types of harmful bacteria. The cell membrane and cell wall are the major defensive boundaries for resisting pathogens in antibacterial actions (Ashraf et al., 2020). MoO_3_ disrupts the bacterial cell, which causes immediate cell death. Its main benefit is that it does not generate bacteria that are resistant to antibiotics. Due to its non-toxicity, it is secure and harmless for human health (Zhao et al., 2020). However, MoO_3_ causes a high recombination rate of electrons and holes. Also, the decrease in the catalytic activity of MoO_3_ was attributed to the self-aggregation of nanoadsorbents, which may reduce the adsorption property and restrict their wide-scale practical application (Li et al., 2022). The addition of dopants (2D materials, rare earth metal or metal oxides, etc.) should be an effective approach for enhancing the properties of MoO_3_. Graphene oxide (GO) gained a lot of attention due to its large surface area and excellent electrochemical and mechanical capabilities. The structure of graphene can effectively prevent nanoparticle aggregation in the liquid-based catalytic process (Yao et al., 2019). The 2D graphitic property of graphene, in contrast to 0D fullerene, 1D carbon nanotubes, and 3D graphite basic components, has been the subject of much research for its photocatalytic properties (Li et al., 2016). These materials have enormous surface areas that give them useful characteristics, including high thermal and mechanical stability and good chemical resistance. To digest hazardous MB in industrially contaminated water, GO and reduced graphene oxide (rGO) are interesting options that may boost the effectiveness of photocatalysis (Xiang et al., 2012). The efficiency of GO/rGO photocatalysts in converting light into chemical energy may be improved by blocking the recombination of electron–hole pairs (Qumar et al., 2021). Polymers may form complexes or ion pairs with metal ions, making them a potential alternative to stabilizers that can be manipulated into achieving the desired results in the physicochemical characterization of NSs (Adzitey et al., 2022). Polymeric materials’ potential uses in biology and ecology have garnered a lot of attention from scientists (NCCLS, 2007). Metal oxide doping is utilized to achieve significant results for a variety of applications, and several polymers (including polyvinyl alcohol, polyvinyl chloride, polyvinylpyrrolidone, and chitosan) are employed (Weidmann et al., 1995; Sampson and de Korte, 2011; Lewis, 2012). PVP, an artificial polymer, is one among them and is widely regarded as an efficient capping material for metal oxide NSs. Carbonyl units and functional groups that reinforce metal oxide NSs are responsible for the composite’s unique characteristics (V Jadhav et al., 2013), (Lu et al., 2012). Because of its high-quality physicochemical qualities, it is employed as a stabilizer for NSs and as an additive in other materials 48–51. PVP has been proven to be biocompatible, minimally toxic, water soluble, and having antibacterial actions that show promise in recent investigations (Shahmiri et al., 2013; Karpuraranjith and Thambidurai, 2017; Hu et al., 2018; Naz et al., 2020; Al Mogbel et al., 2021). Numerous methods (hydrothermal, sol–gel, thermal deposition, co-precipitation, physiochemical techniques, etc.) can be used to prepare MoO_3_ NSs. Co-precipitation is the most practical technique due to its low cost and eco-friendly nature (Ikram et al., 2022). Among the several suggested approaches, co-precipitation has the characteristics of being easily repeatable, low in impurity, and easily processable (Naz et al., 2020). As direct precipitation is unable to separate the desired metallic species because of its low concentration in the sample solution, co-precipitation can be adopted. It is less time consuming, uses basic laboratory equipment that makes it easily processable, and is a low-cost technique (Bader et al., 2014). The goal of this study is to synthesize GO/PVP-doped MoO_3_ NSs via the co-precipitation method for the breakdown of organic dyes from contaminated water and to examine their bactericidal effect against *E*. *coli*. As PL intensity decreases, the electron charge efficiency increases, which leads to the generation of reactive oxygen species that may produce more active sites resulting in good antibacterial activity The majority of bacterium cell walls (including those of *E. coli* and *S. aureus*) are known to be negatively charged. In fact, due to an electrostatic interaction with a negatively charged bacterium surface, some chemicals (quaternary ammonium compounds, molecules, and polymers) can trigger membrane rupture and, ultimately, lead to subsequent death. Active substances are more likely to bind and be transferred through the skin if they are able to construct a hydrogen-bonded network with water molecules, which is facilitated by the existence of hydroxyl groups (Kupnik et al., 2020).

## 2 Experimental section

### 2.1 Materials

Ammonium molybdate (NH_4_)_6_ Mo_7_O_24_.4H_2_O and PVP (C_6_H_9_)_n_ were purchased from Sigma-Aldrich, Germany.

### 2.2 Synthesis of GO

**FIGURE 1:**
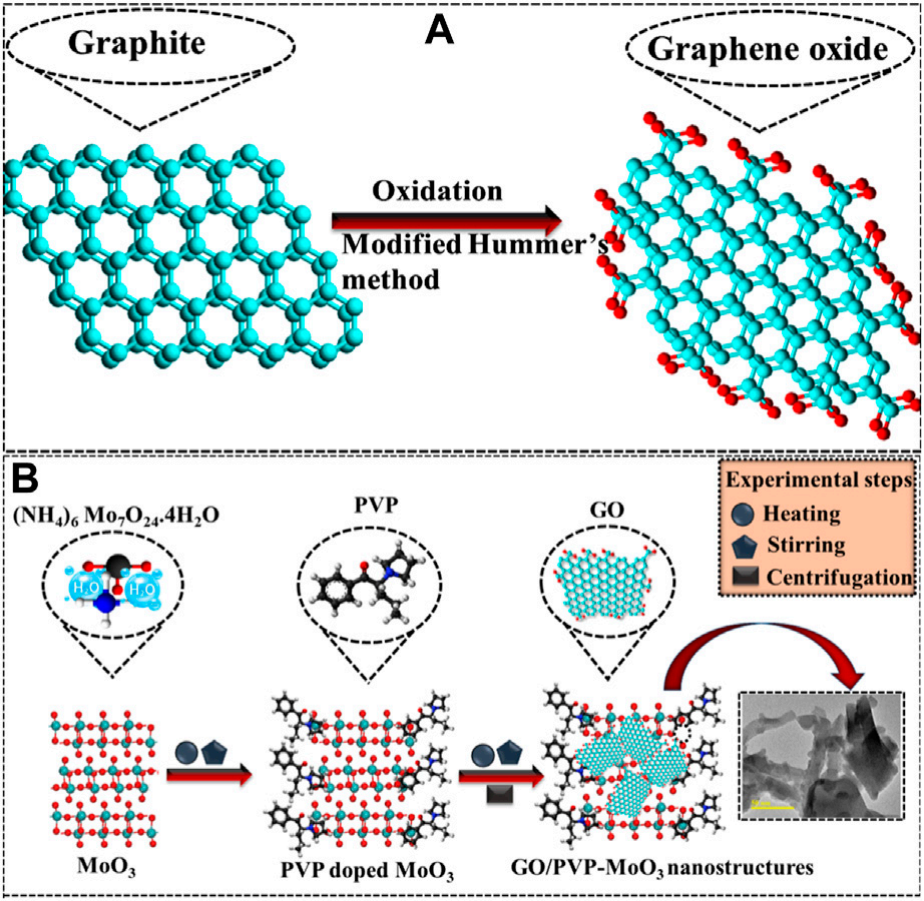
**(A)** Synthesis of GO; **(B)** synthesis of (2 and 4 wt%) GO/PVP-doped MoO_3_ NSs.

Refined graphite was used to synthesize GO via the modified Hummers technique. Graphite (5 g) and NaNO_3_ (2.5 g) were integrated into H_2_SO_4_ (108 mL) with H_3_PO_4_ (12 mL), and the solution was vigorously stirred for 10 min in a reaction flask (immersed in an ice bath). The filtrate solution was poured into a muffle furnace for 2 h (60°C) to remove moisture. Later, KMnO_4_ (15 g) was incorporated slowly, while the temperature was kept below 5°C and vigorously stirred; the solution color turned from purple to yellow. After the addition of H_2_O_2_ (12 mL), the suspension was then centrifuged (7,000 rpm) and repeatedly washed with deionized water (DI water) to achieve a residue. Afterward, the resulting precipitates were dried for 12 h and ground to get a fine powder (Figure 1A).

### 2.3 Synthesis of GO/PVP-doped MoO_3_

To synthesize MoO_3_ by the co-precipitation technique, 0.1 M of ammonium molybdate was prepared under continuous stirring at 90°C for 40 min. The desired amount of HCl was added dropwise in the aforementioned solution to keep the pH ∼2. Afterward, the colloidal solution was centrifuged two times (7,000 rpm front 7 min) with DI water, dried for 12 h at 150°C, and ground to attain a fine powder. Similarly, different concentrations of GO (2 and 4 wt%) and fixed amounts of PVP-doped MoO_3_ NSs were prepared (Figure 1B).

### 2.4 Catalysis (CA)

The catalytic efficacy of the host and doped MoO_3_ for the reduction of rhodamine B (RhB) was analyzed in the presence of sodium borohydride (NaBH_4_). First, 0.1 M NaBH_4_ was added into the RhB solution followed by the integration of 200 μL of MoO_3_ and GO/PVP-doped MoO_3_. The change of the color of the dye from pink to light pink indicated the reduction of RhB into leuco-rhodamine B (LRhB). The obtained supernatant was examined using a UV–Vis spectrometer to measure the degree of dye degradation in a periodic time interval. The percentage of dye degradation for each sample was computed via the followingequation:

$$\%\;Degradation=C_{o}-C_{t}/C_{o}\times 100,$$ (1)

where C_o_ is the initial concentration of the dye at t_0_ and C_t_ is the final concentration of the dye at t after the incorporation of composite materials.

### 2.5 Isolation and identification of MDR *E. coli*

#### 2.5.1 Sample collection

The selected nursing cows were milked directly into sterilized glassware, available at several markets, veterinary clinics, and farms in Punjab, Pakistan. After being collected at 4°C, raw milk was immediately brought to the lab. We examined the number of coliforms in raw milk on MacConkey agar. 48 h were spent incubating all the plates at 37°C.

#### 2.5.2 Identification and characterization of bacterial isolates

Using Bergey’s Manual of Determinative Bacteriology as a reference, the first identification of *E. coli* was based on the colonial morphology detected after Gram staining and many biochemical tests (Sinclair, 1939).

#### 2.5.3 Antibiotic susceptibility

The Bauer disc diffusion method was used to perform the antibiotic susceptibility test on Mueller Hinton agar (MHA) (Bauer et al., 1966). The test was performed to check whether *E. coli* was resistant to the following antibiotics (classes): azithromycin (Azam) 15 µg (macrolides), gentamicin (Gm) 10 µg (aminoglycosides), ciprofloxacin (Cip) 5 µg (quinolones), amoxicillin (A) 30 μg (penicillin), tetracycline (Te) 30 µg (tetracyclines), imipenem (Imi) 10 µg (carbapenem), and ceftriaxone (Cro) 30 µg (cephalosporins) (Adzitey et al., 2022). *E. coli* purified cultures were grown and adjusted to 0.5 McFarland turbidity. The spread plate was placed on MHA (Oxoid Limited, Basingstoke, United Kingdom). The antibiotic discs were set away from the inoculation plate to prevent the overlap of inhibition zones. The Clinical and Laboratory Standards Institute evaluated the results after the plates were incubated at 37°C for 24 h (NCCLS, 2007). MDR was given to bacteria that were discovered to be resistant to at least three antibiotics (Iwalokun et al., 2004).

### 2.6 Antimicrobial activity

Using the agar well diffusion method, the *in vitro* antibacterial activity of MoO_3_ was assessed against 10 typical isolates of MDR *E. coli* obtained from mastitic milk. Petri dishes were swabbed with 1.5 × 10^8^ CFU/mL (0.5 McFarland standard) MDR *E. coli* on MacConkey agar. Using a sterile cork borer, 6 mm-diameter wells were created. 2 and 4% GO/PVP-MoO_3_ were applied as 500 μg/0.05 mL and 1,000 μg/0.05 mL, respectively. Ciprofloxacin (5 μg/0.05 mL) was used as the positive control and DI water as the negative control (0.05 mL) (Haider et al., 2020a).

#### 2.6.1 Statistical analyses

The inhibition zone (mm) size was used to detect the antimicrobial efficiency and was analyzed statistically by one-way analysis of variance (ANOVA) using SPSS 20 (Haider et al., 2020b)

### 2.7 Molecular docking analysis

Enzyme targets involved in fatty acid and folate biosynthesis pathways, specifically enoyl-[acyl carrier protein] reductase (FabI) and dihydrofolate reductase (DHFR) from *E. coli*, were subjected to molecular docking. DHFR (PDB ID: 2ANQ; resolution: 2.13 Å) (Summerfield et al., 2006) and FabI (PDB ID: 1MFP; resolution: 2.33 Å) (Seefeld et al., 2003) have been acquired from the Protein Data Bank. SYBYL-X 2.0 (Mehmood et al., 2022) was used to predict the results of the molecular docking analysis. The SYBYL-X 2.0/SKETCH module was used to create 3D structures of selected compounds, followed by energy minimization using the Tripos force field with the Gasteiger–Hückel atomic charge (Clark et al., 1989). The Surflex–Dock module of molecular modeling software program SYBYL-X 2.0 (Shahzadi et al., 2023) was used to analyze flexible molecular docking simulations to study binding interactions of nanoparticles with active site residues of the selected proteins. The missing hydrogens were introduced. According to the AMBER 7 ff99 force field, the atomic types were allocated and atomic charges were applied. Finally, using the Powell algorithm with a convergence gradient of 0.5 kcal/(molA) for 1,000 cycles, the energy was reduced to avoid steric clashes. For each ligand-receptor complex system, at least 20 of the finest docked poses were saved conclusively. The Hammerhead scoring system was used to rate the best putative ligand poses. The Surflex–Dock module generates and ranks putative poses of ligand fragments using an empirically generated consensus scoring (cScore) (Blount et al., 2002) function that combines Hammerhead’s empirical scoring function (Jain, 1996), comprised of the D score (dock score), G score (gold score), ChemScore, potential mean force (PMF) score, and/or complete score, with a molecular similarity method (morphological similarity).

## 3 Results and discussion

The modified Hummers method was used to prepare GO, and (2 and 4 wt%) GO/PVP-doped MoO_3_ was obtained using the co-precipitation technique.

**FIGURE 2:**
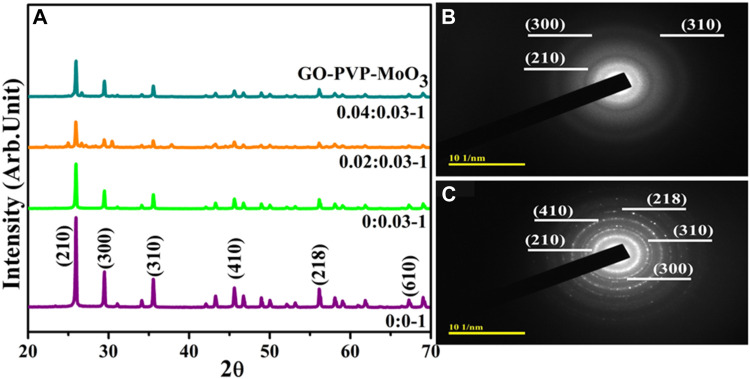
**(A)** XRD patterns; **(B,C)** SAED pattern of (2 and 4 wt%) GO/PVP-doped MoO_3_.

The XRD analysis was used to investigate the phase purity, crystalline structure, and inter-planar properties of pure and GO/PVP-doped MoO_3_, as shown in Figure 2A. Diffraction peaks were observed at 25.84°, 29.46°, 35.52°, 45.57°, 56.19°, and 67.25°, along with matching crystal planes, (210), (300), (310), (410), (218), and (610), respectively. Furthermore, the hexagonal crystal structure of MoO_3_ was confirmed by JCPDS file no. 00-021-0569. Minor shifting toward a lower angle can be seen after doping. The crystallinity of prepared NSs was reduced by the incorporation of GO, manifested for anchoring of MoO_3_ on nanosheets (Munawar et al., 2022). Additionally, the SAED analysis of MoO_3_ and GO/PVP-doped MoO_3_ exhibited circular rings corresponding to various planes, (310), (300), (210), (218), and (410), of XRD (Figures 2B, C).

**FIGURE 3:**
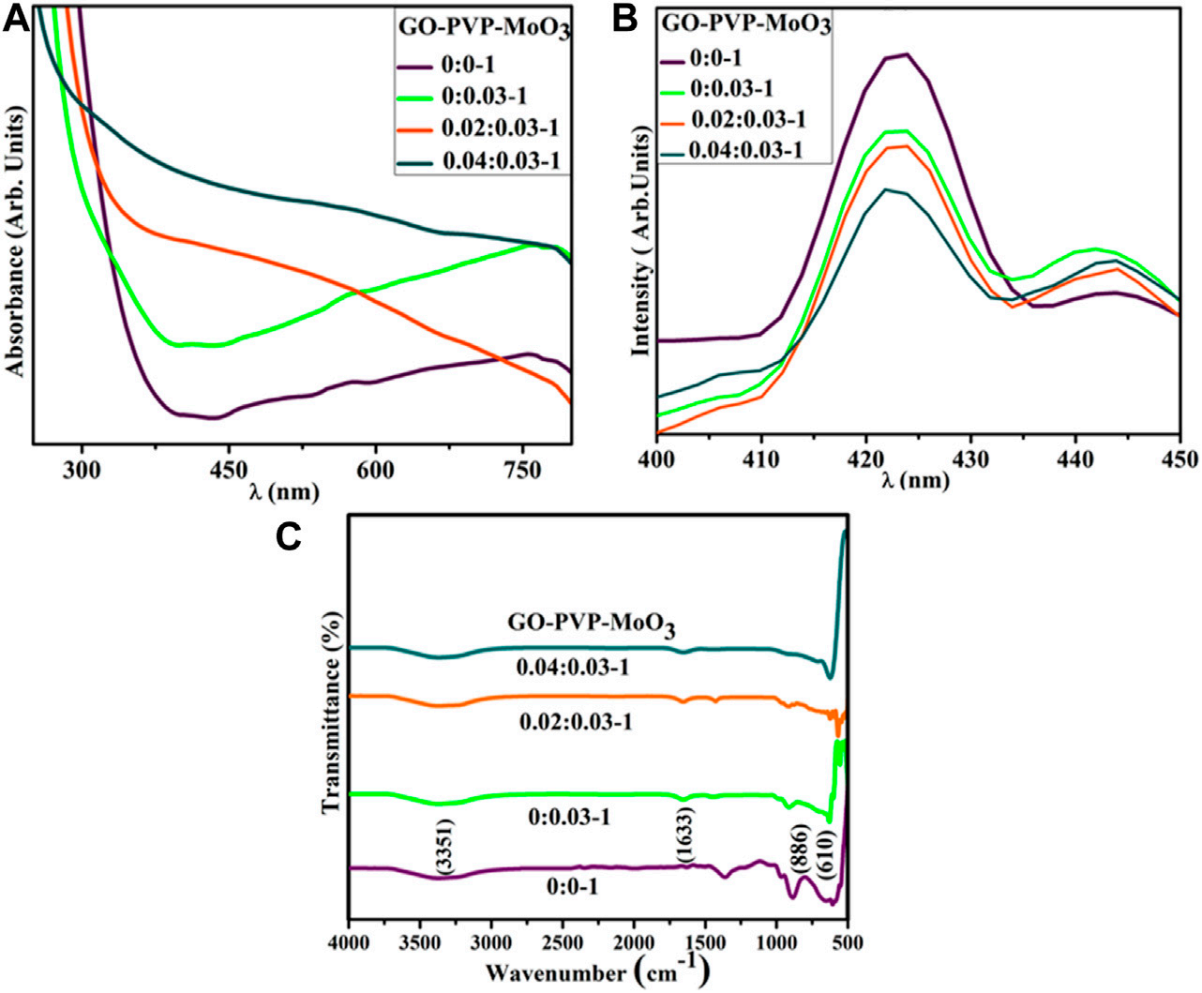
**(A)** UV–Vis spectra, **(B)** PL-spectra, and **(C)** FTIR spectra of (2 and 4%) GO/PVP-doped MoO_3_.

UV–Vis spectroscopy was employed to examine the optical characteristic of pure and (2 and 4 wt%) GO- and PVP-doped MoO_3_ (Figure 3A). MoO_3_ exhibited the absorption range between 300 and 340 nm (Gowtham et al., 2018). Upon doping of GO and PVP, a red shift was observed that led to the decrease in the band gap energy, which might be the quantum confinement effects. The band gap of pure MoO_3_ NSs was calculated to be 2.7 eV, which decreases upon doping. PL spectroscopy was employed to investigate the optical characteristics, quantum confinement processes, and energy levels of the material. A photoluminescence signal is produced when electrons in the valence band (VB) are excited with the conduction band (CB) at an excitation wavelength and then returned to the VB. MoO_3_ NSs yield broad emission peaks in the visible range from 415 to 430 nm at an exciton wavelength of 300 nm (Klinbumrung et al., 2012). Upon doping of GO and PVP, the intensity of MoO_3_ was reduced, attributed to a decrease in the exciton recombination rate (Figure 3B).

The FTIR technique was used to investigate the existence of the functional group in a host and doped MoO_3_, with a wavenumber range of 4,000–500 cm^-1^ (Figure 3C). The transmittance band at 610 cm^−1^ was assigned as O–Mo–O stretching and bending vibrations (Song et al., 2007), and the band at 886 cm^−1^ in the range of 875–885 cm^−1^ was attributed to Mo–O–Mo vibrations of Mo (Chiang and Yeh, 2013). The bands at 1,633 and 3,351 cm^−1^ were manifested for the bending and stretching of the absorbed hydroxyl function group (Bari et al., 2022).

**FIGURE 4:**
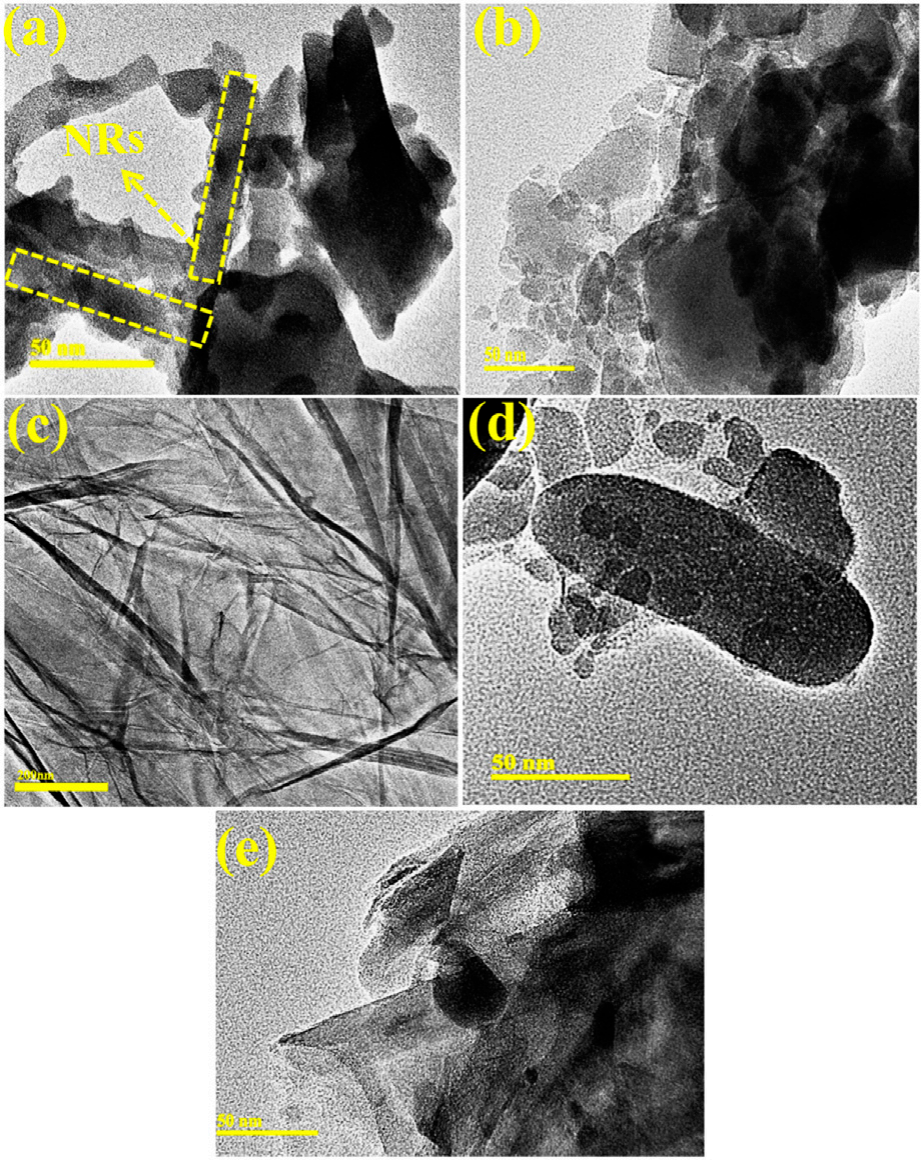
**(A–E)** TEM images of **(A)** MoO_3_, **(B)** PVP–MoO_3_, **(C)** GO, **(D)** 2% GO/PVP–MoO_3,_ and **(E)** 4% GO/PVP–MoO_3_.

EDS analysis was performed to evaluate the elemental composition of the pure and (2 and 4 wt%) GO/PVP-doped MoO_3_. Intense peaks of Mo and O confirmed the purity of the synthesized material, as shown in Supplementary Figure S1. Gold (Au) peaks can be seen in the spectra because the sample has a Au coating applied to it to reduce the influence of charge. Small Cu peaks may have been caused by the brass sample holder used for the EDS observation. TEM was used to determine the morphological properties of the prepared samples, as shown in Figures 4A–E. MoO_3_ showed randomly oriented nanorod-like (NR) structures with nanoparticles, as demonstrated in Figure 4A. The addition of PVP into MoO_3_ showed an aggregation of nanoparticles (Figure 4B). Figure 4C confirmed the nanosheets of pristine GO, and the incorporation of GO in the binary system (PVP–MoO_3_) showed that GO nanosheets overlapped the nanoparticles and agglomeration increased with the increase in the amount of GO (Figure 4D). Using Gatan software, interlayer d-spacing was determined from HRTEM images, as shown in Supplementary Figure S2. The d-spacing for GO/PVP-doped MoO_3_ was recorded as 0.20, 0.16, 0.25, and 0.13 nm, which was well matched with the XRD results.

**FIGURE 5:**
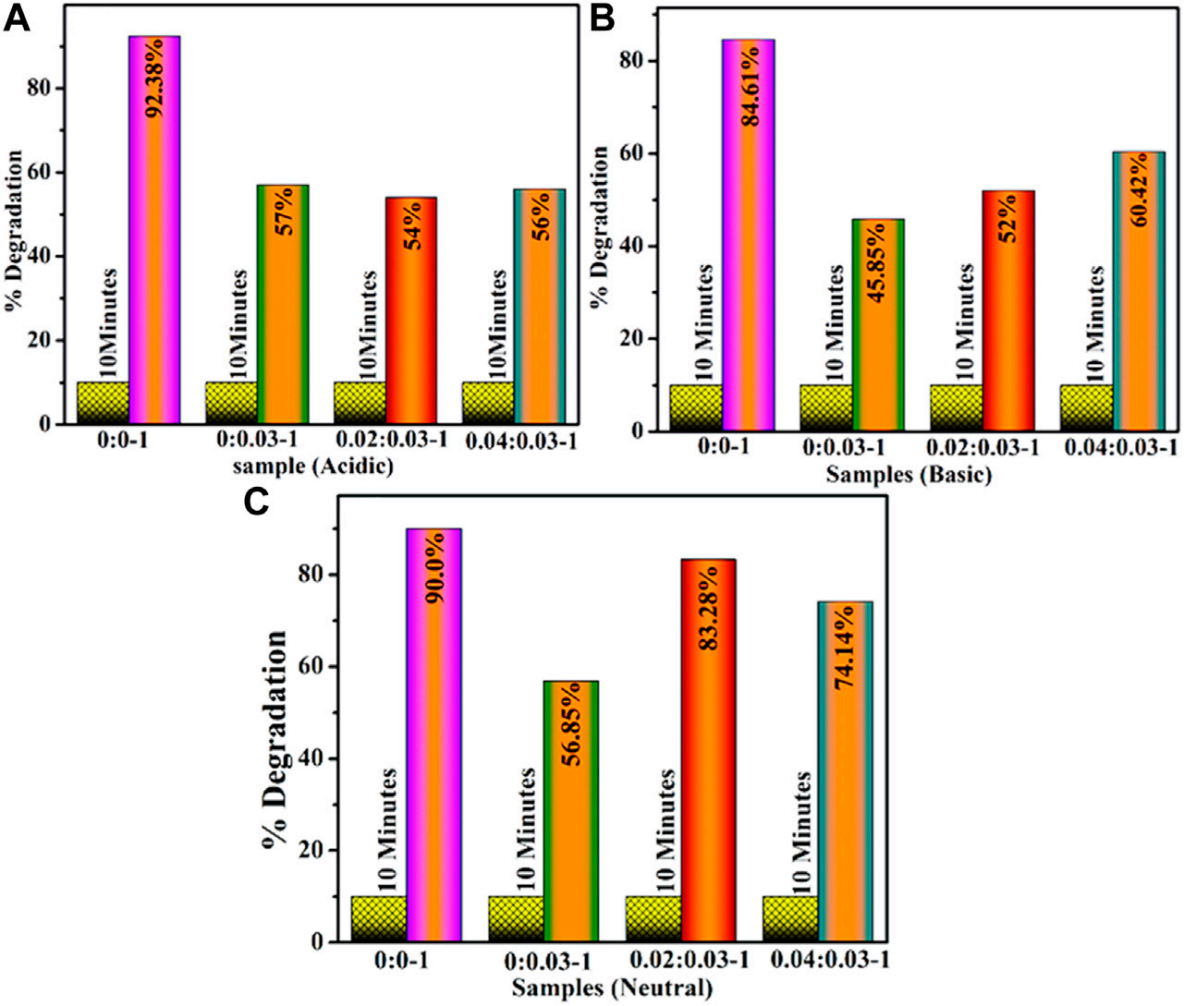
Catalytic activity of (2 and 4 wt%) of GO- and PVP-doped MoO_3_ in **(A)** acidic, **(B)** basic, and **(C)** neutral media.

The degradation efficacy of dopant-free and (2 and 4 wt%) GO/PVP-doped MoO_3_ was investigated for a time interval of 10 min for acidic values of 92.38, 57, 54, and 56%, shown in Figure 5A, for basic values of 84.61, 45.85 52, and 60.42%, shown in Figure 5B, and for neutral values as 90.0, 56.85, 83.28, and 74.14%, shown in Figure 5C. In all media, MoO_3_ NSs resulted in the highest concentrations of catalytic activity, while the addition of dopants decreased the efficiency of catalysis. The decreased degradation efficiency of dopants may be due to the self-aggregation of the nanoadsorbents, which may reduce the adsorption property and restrict their wide-scale practical application (Li et al., 2022).

The two main aspects that are thought to be crucial in the catalysis mechanism are adding a nanocatalyst and a reducing agent to the dye (Jana and Pal, 1999; Panigrahi et al., 2007; Khalavka et al., 2009; Maerzke and Siepmann, 2011). As nanoparticles have a large surface area, MoO_3_ NSs serve as an electron relay system for dye catalytic reduction (Hu et al., 2007). The catalytic process for reducing RhB from MoO_3_ NSs in the presence of BH^−^_4_ ions is shown in Supplementary Figure S3. Initially, BH^−^_4_ and RhB will absorb over a large surface area of MoO_3_ NSs. In general, BH^−^_4_ ions act nucleophilic and give electrons to MoO_3_, while RhB acts electrophilic and can take electrons away from MoO_3_ to generate a reduced form of leuco RhB. Leuco RhB and BH_4_ have been desorbed from the MoO_3_ NS surface. Because of their large surface area, sodium borohydride and MoO_3_ NSs must be loaded more frequently. The reaction rate of RhB degradation considerably increases when the concentration of MoO_3_ NSs rises (Cazaux and Tielens, 2004). A comparison of the antibacterial activity of the synthesized nanostructures in previous studies with the results of the current investigation (Table 2).

**FIGURE 6:**
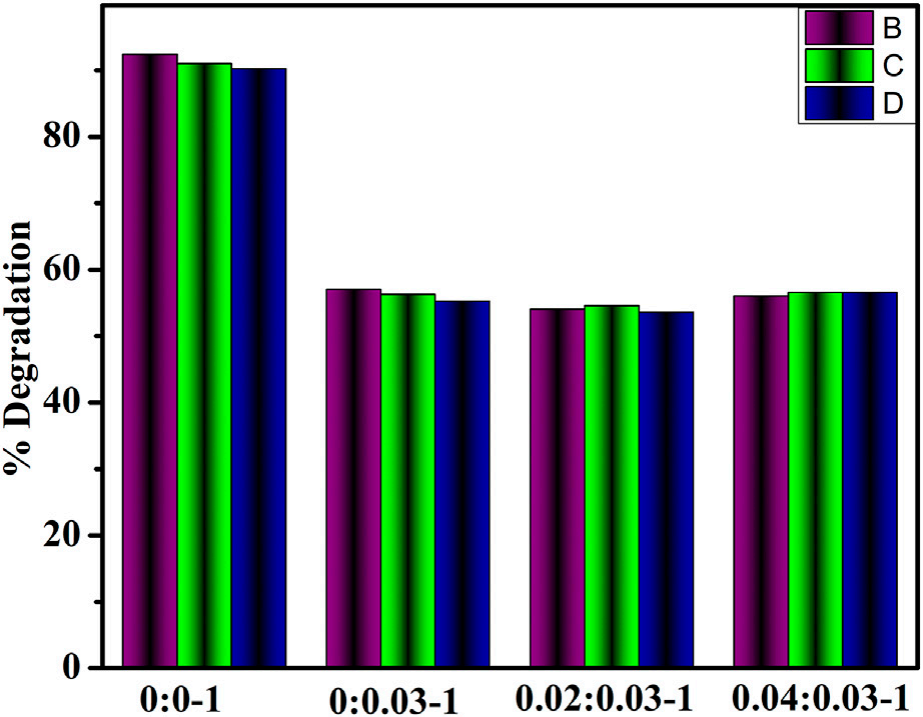
Stability of (2 and 4 wt%) GO/PVP-doped MoO_3_ in an acidic medium.

The stability of GO/PVP-MoO_3_ was tested by storing the degraded solution samples in the dark for 3 days to see if the dye degradation was stable. Dye degradation efficiency was monitored via UV–Vis spectrophotometry every 24 h, as shown in Figure 6. Using Eq. 1, the efficiency of percentage degradation was calculated.

The antibacterial behavior of GO/PVP-doped MoO_3_ is summarized in Table 1. Undoped MoO_3_ showed less antibacterial activity (Naseem and Durrani, 2021), while GO/PVP-doped MoO_3_ has an increased bactericidal action. *E. coli* showed an inhibitory zone at low and high doses ranging from (2.65–6.90) to (4.05–8.65). In addition, the ciprofloxacin inhibition zone against *E. coli* was measured as 9.65 mm (positive control), parallel to 0 mm of DI water (negative control). Oxidative stress produced by nanomaterials is proportional to their concentration, size, and shape. The antibacterial efficiency of the substance is inversely associated with its size. GO/PVP-doped MoO_3_ produces more reactive oxygen species, called ROS, due to its smaller size (Yu et al., 2020). These species, in turn, cause the extrusion of cytoplasmic components as seen from Supplementary Figure S4, which ultimately leads to the death of bacteria by the penetration of the micro-organism membrane. Establishing oxidative stress effectively supplements the fundamental antibacterial function, which is accomplished by intimate contact with GO and involves a non-oxidative electron exchange procedure triggered by interactions between GO and the metal substrate. The reactive oxygen species (e.g., H_2_O_2_) produced by GO have the potential to impair oxygen consumption, energy transduction, the energetic equilibrium of phospholipids, and the transport of physiologically active molecules, all of which might lead to serious structural degradation of cell membranes (Haider et al., 2020b).

**TABLE 1:** Antibacterial activity of (2 and 4 wt%) GO/PVP-doped MoO_3_.

Samples	*E. coli*		Inhibition zone (mm)	
	500 μg/0.05 mL	1,000 μg/0.05 mL	Ciprofloxacin	Deionized water
MoO_3_	2.65	4.05	9.65	0
PVP/MoO_3_	5.75	7.25	9.65	0
2% GO/PVP/MoO_3_	6.15	8.15	9.65	0
4% GO/PVP/MoO_3_	6.90	8.65	9.65	0

**TABLE 2:** Literature comparison of the antibacterial activity of synthesized NSs with the present study.

Nanocatalyst	Synthesis route	Antibacterial activity (*E. coli*)	References
h-MoO_3_	Chemical bath deposition	0	Desai and Mali (2015)
MoO_3_	Epigallocatechin gallate-mediated approach	0.25	Yogananda et al. (2018)
MoO_3_	Ball milling approach	8	Krishnamoorthy et al. (2014)
MoO_3_	Wet chemical approach	8	Krishnamoorthy et al. (2013)
GO/PVP–MoO_3_	Co-precipitation	8.65	Present work

Several researchers (Seefeld et al., 2003; Summerfield et al., 2006; Mehmood et al., 2022) have investigated the microbicidal capability of metal ion-containing nanocomposites (Clark et al., 1989), (Shahzadi et al., 2023). The bioactivity of the nanocomposites depends on their tendency to interface bacteria via electrostatic, van der Waals, or hydrophobic forces. The enzymes associated with essential metabolic activities in bacterial metabolic processes have been identified, potentially promising antibiotic candidates. Thus, the fatty acid biosynthesis enzyme FabI and the folate biosynthesis enzyme DHFR from *E. coli* were adopted for viable species to examine the inhibitory response of PVP/MoO_3_ with GO/PVP/MoO_3_ against them.

**FIGURE 7:**
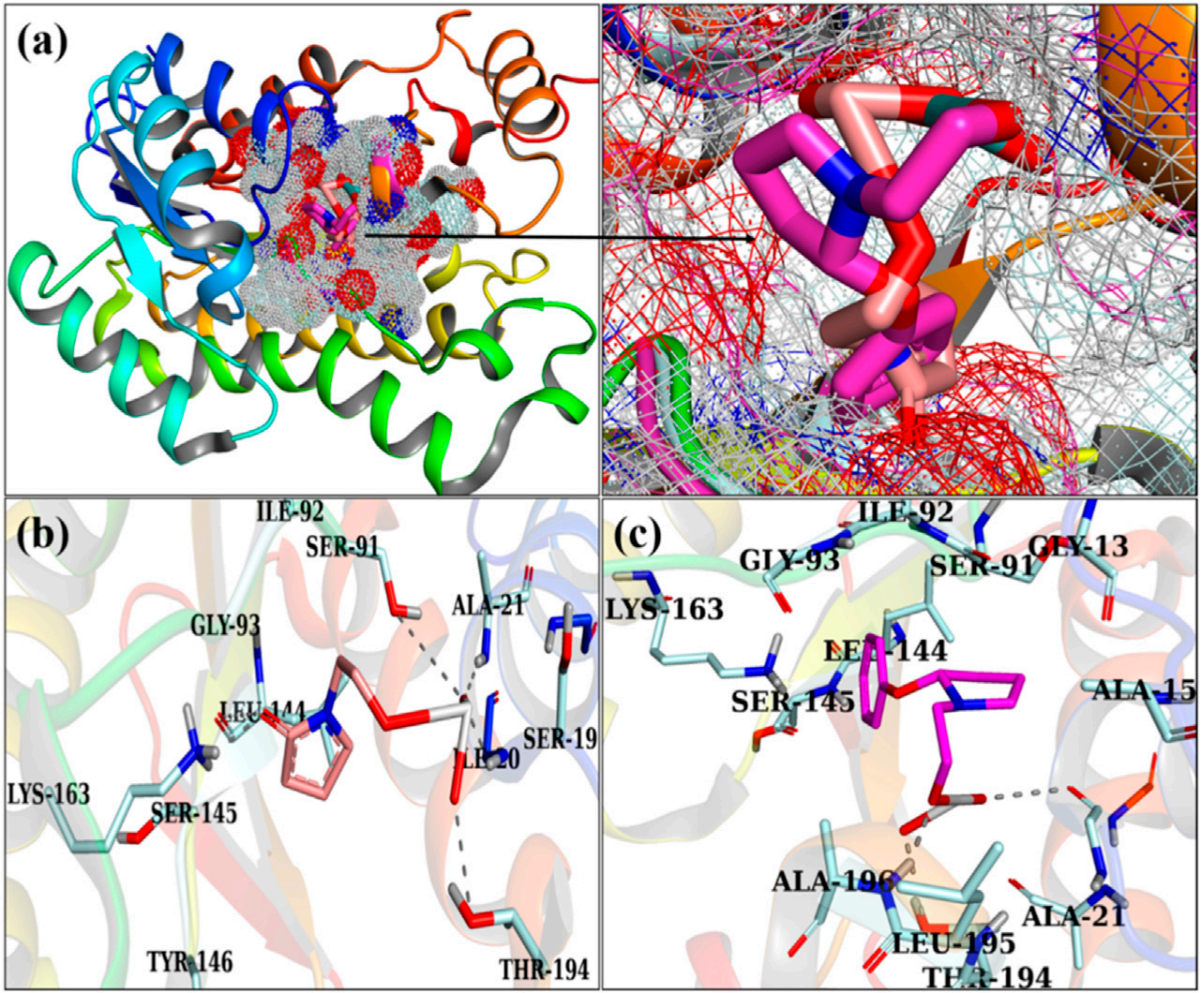
3D view of a binding pocket **(A)** and the binding interaction pattern of PVP/MoO_3_ **(B)** and GO/PVP/MoO_3_ **(C)** inside the binding site of FabI.

Figures 7A, B illustrate the optimally docked conformation of PVP/MoO_3_ within the active domain of FabI *E. coli*, which disclosed an H-bond with Ile20, Ala21, Ser41, Lys163, and Thr194 and a total binding score of 5.05. The key binding contacts for GO/PVP/MoO_3_ were Ser19, Thr194, and Ala196, respectively, with a binding score of 7.20, as shown in (Figures 7A–C).

**FIGURE 8:**
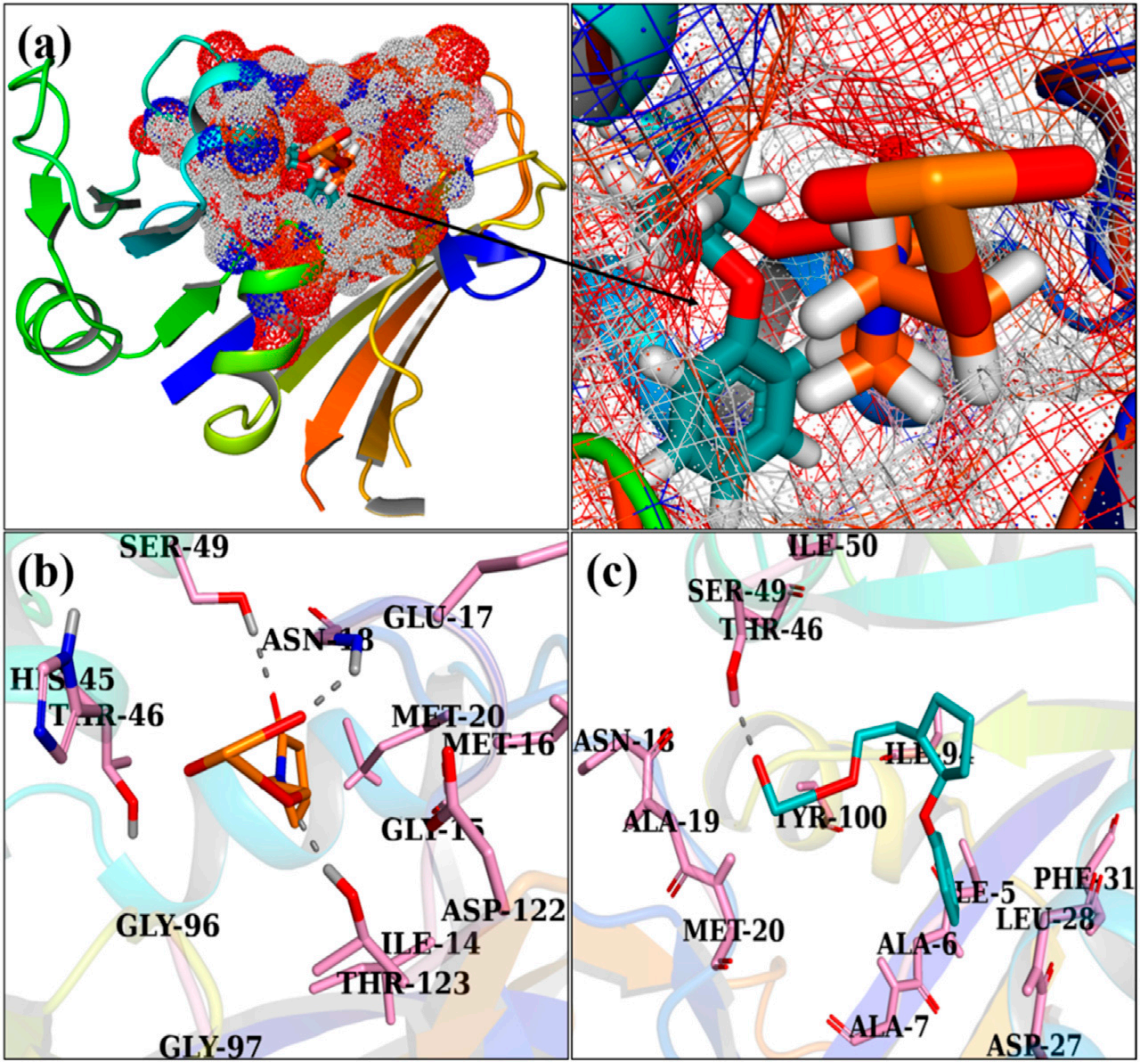
3D view of a binding pocket **(A)** and the binding interaction pattern of PVP/MoO_3_ **(B)** and GO/PVP/MoO_3_ **(C)** inside the binding site of FabI.

Figures 8A, B exhibit the optimally docked conformation of PVP/MoO_3_ in the active pocket of DHFR *E. coli*, which exhibited an H-bond interaction with Asn18, Ser49, and Thr123 and a total binding score of 3.91. The primary binding interactions for GO/PVP/MoO_3_ were with Ser49, which had a binding score of 5.91 (Figures 8A–C).

## 4 Conclusion

In this study, GO/PVP-doped MoO_3_ NSs were effectively synthesized using a low-cost co-precipitation method. The XRD pattern confirmed the presence of the hexagonal structure of synthesized NSs. A rod-like morphology of MoO_3_ was recorded by TEM, while a higher concentration of GO revealed the encapsulation of nanorods to nanosheets. FTIR has confirmed the existence of O–Mo–O stretching vibrations of the synthesized NSs. Moreover, EDS spectra have confirmed the presence of Mo, PVP, and GO. UV–Vis spectroscopy revealed absorption peaks for MoO_3_ and GO/PVP-doped MoO_3_; however, the red shift was observed due to the quantum confinement effect. Compared to the doped samples, the undoped MoO_3_ nanostructure showed the highest catalytic potential. Additionally, the prepared nanostructures were highly efficient in inhibiting *E. coli*. In conclusion, MoO_3_ NSs with natural and synthetic polymers may be inexpensive and effective against microbes, but they are resistant to the degradation of industrial dyes. This study suggests that 4% GO/PVP-doped MoO_3_ NSs could be used as effective antibacterial agents against *E. coli* (Dakal et al., 2016; Altaf et al., 2020; Arularasu et al., 2020; Ikram et al., 2020; Shahzadi et al., 2022).

## Data Availability

The original contributions presented in the study are included in the article/Supplementary Material; further inquiries can be directed to the corresponding authors.
