# Supplementary material for: Graphene oxide/polyvinylpyrrolidone-doped MoO3 nanocomposites used for dye degradation and their antibacterial activity: a molecular docking analysis
Source: Front Chem. 2023 May 9;11:1191849. doi: 10.3389/fchem.2023.1191849 (PMC10205020; doi:10.3389/fchem.2023.1191849)
Supplement: Supplementary file 1 [file DataSheet1.docx]

**Supplementary Materials**

**Graphene oxide/polyvinylpyrrolidone doped MoO_3_ nanocomposites used for dye degradation and their antibacterial activity: Molecular docking analysis**

Muhammad Ikram^a*^, Iram atiq^b^, Alvina Rafiq Butt^b^, Iram shahzadi^c^, Anwar Ul-Hamid^d^, Ali Haider^e*^, Walid Nabgan^f^, Junaid Haider^g^

^a^Solar Cell Applications Research Lab, Department of Physics, Government College University Lahore, Lahore, 54000, Punjab, Pakistan

^b^Department of Physics, Lahore Garrison University, Lahore, 54000, Punjab, Pakistan

^c^Punjab University College of Pharmacy, Allama Iqbal Campus, University of the Punjab, Lahore, Pakistan

^d^Core Research Facilities, Research Institute, King Fahd University of Petroleum & Minerals, Dhahran, 31261, Saudi Arabia

^e^Faculty of Veterinary and Animal Sciences, Muhammad Nawaz Shareef University of Agriculture, Multan, 66000, Punjab, Pakistan

^f^Departamentd’Enginyeria Química, Universitat Rovira i Virgili, Av Països Catalans 26, 43007, Tarragona, Spain

^g^Tianjin Institute of Industrial Biotechnology, Chinese Academy of Sciences, Tianjin 300308, China

*Corresponding authors email: [dr.muhammadikram@gcu.edu.pk](about:blank) (M. Ikram), [ali.haider](about:blank)@mnsuam.edu.pk (A. Haider), [wnabgan@gmail.com](mailto:wnabgan@gmail.com) (W. Nabgan)


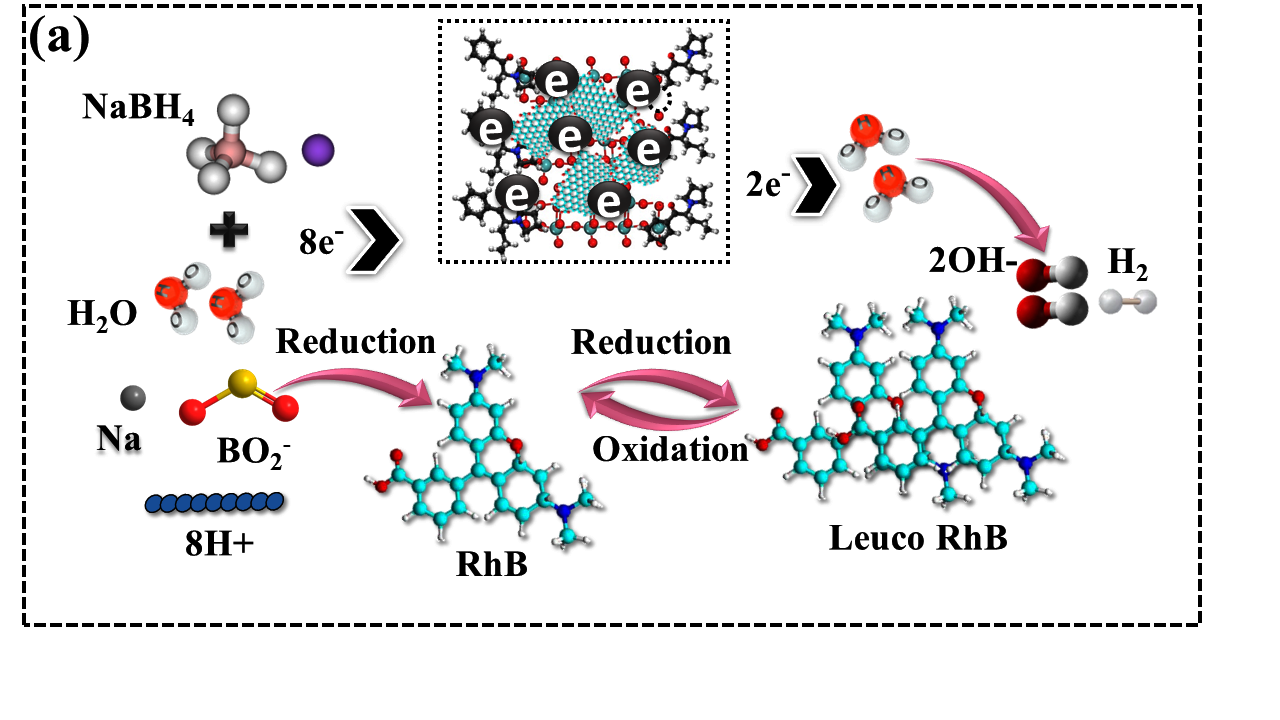
 **Figure. S1:** Catalysis mechanism of prepared nanostructures


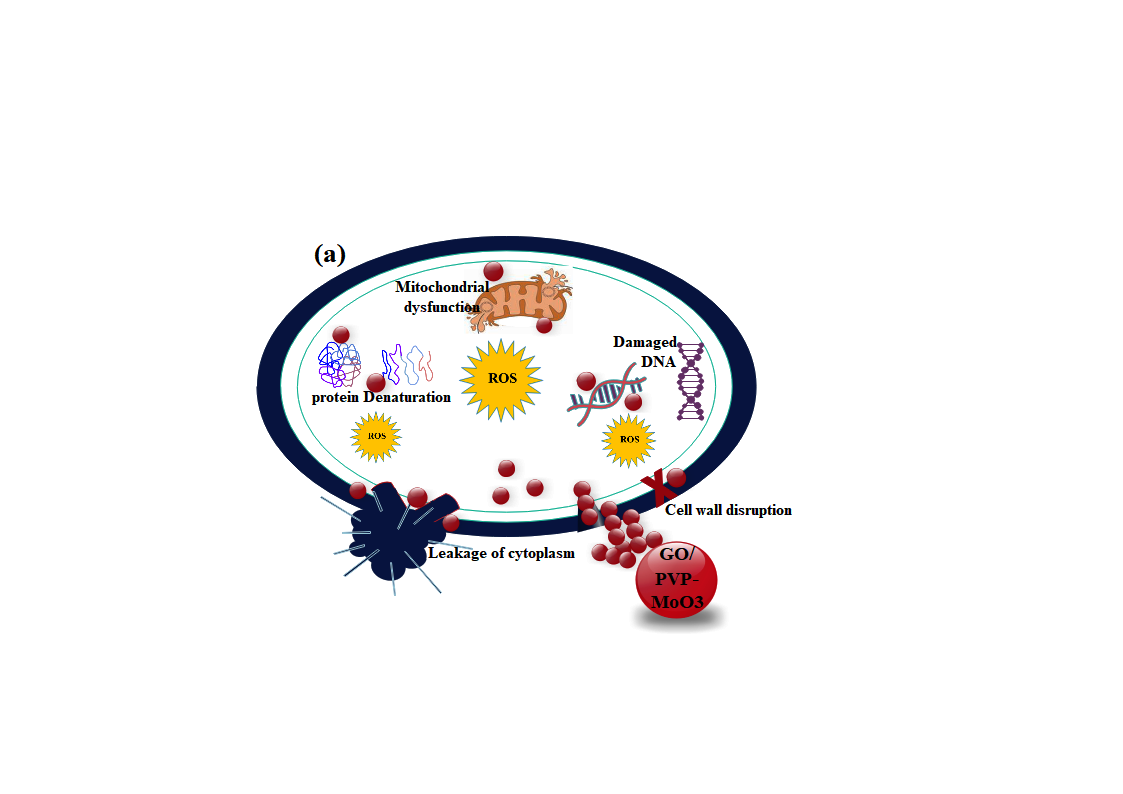


 **Figure. S2:** Bactericidal behavior of (2, 4%) GO/PVP doped MoO_3


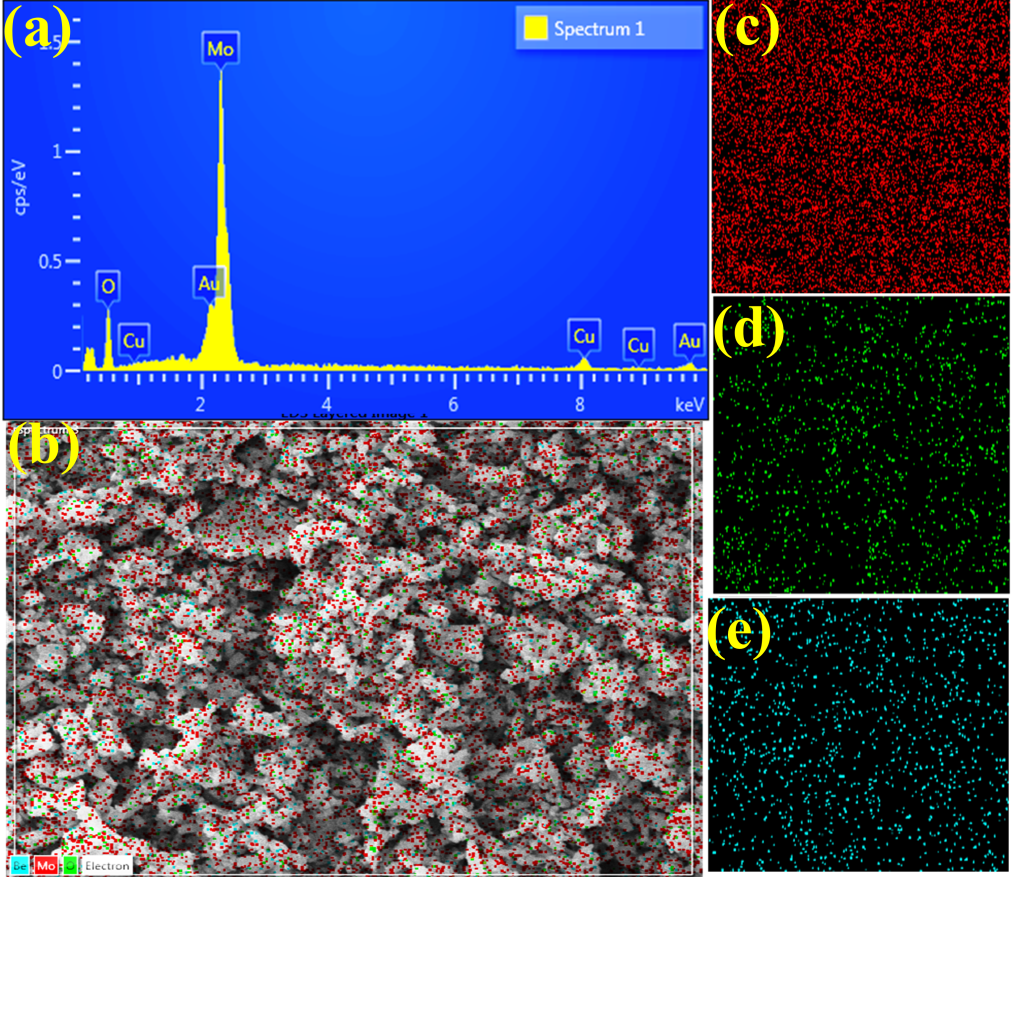
_**Figure. S3:** EDS patterns of (2, 4%) GO/PVP -doped MoO_3_ and **(b-e)** represent the concentration of Mo, O, Be.

**
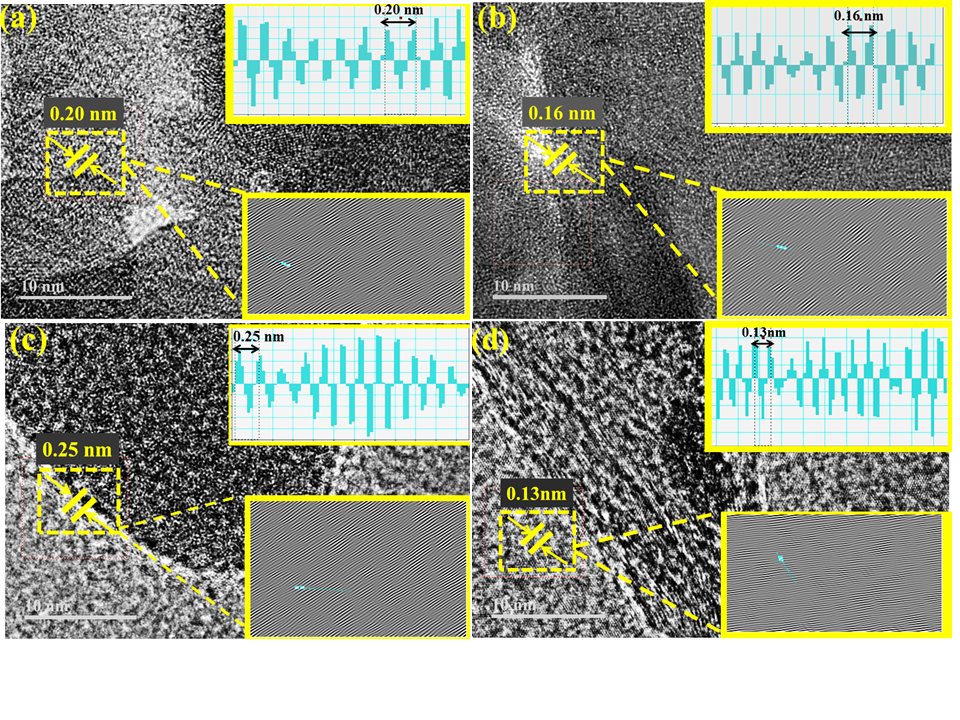
**
 **Figure. S4:** d- spacing of (2, 4%) GO/PVP -doped MoO_3_
